# Tyrer–Cuzick Lifetime Risk Is Not Associated With Non‐BRCA1/2 Pathogenic Variants for Breast Carcinoma

**DOI:** 10.1155/tbj/8670441

**Published:** 2026-01-19

**Authors:** Divya Rao, Chloe Wernecke, Lisa Baron, Steven Cai, Peter Beitsch, Rakesh Patel, Pat Whitworth, Barry Rosen, Nhan Nguyen Tran, Kevin Hughes, Paul L. Baron

**Affiliations:** ^1^ Department of Emergency Medicine, Nassau University Medical Center, East Meadow, New York, USA, nuhealth.net; ^2^ Department of Surgery, Lenox Hill Hospital, Northwell Health, New York, New York, USA, northwell.edu; ^3^ Targeted Medical Education, Dallas, Texas, USA; ^4^ Dallas Surgical Group, Dallas, Texas, USA; ^5^ Department of Radiation Oncology, Good Samaritan Hospital, Los Gatos, California, USA, goodsam.org; ^6^ Nashville Breast Center, Nashville, Tennessee, USA; ^7^ Advocate Aurora Healthcare, Downers Grove, Illinois, USA; ^8^ Department of Surgery, Medical University of South Carolina, Charleston, South Carolina, USA, musc.edu

**Keywords:** breast cancer, risk model, Tyrer–Cuzick

## Abstract

**Background:**

The Tyrer–Cuzick (TC) or IBIS risk calculator is a widely used tool to estimate the probability of developing breast cancer. The latest version incorporates various factors to assess the risk of breast cancer, including family history, personal history, breast density, and past medical history. The TC is commonly used to guide patients toward further diagnostic imaging, genetic testing, chemoprevention, or risk‐reducing surgery. However, it is unclear whether the TC is associated with non‐BRCA1/2 pathogenic variants (PVs) in breast cancer susceptibility genes.

**Methods:**

A population of 964 patients with TC was evaluated for 12 PVs and variants of unknown significance (VUS) using lab‐agnostic genetic testing. Patients were enrolled from 2019 to 2022. Historical TC were used for the subgroup of patients who developed breast cancer after enrollment. TC scores were compared between the three patient cohorts that had BRCA gene mutations, non‐BRCA PVs, and negative for PVs, using the Kruskal–Wallis test followed by pairwise comparison using DSCF adjustment for multiple comparisons. Data collection for patient cohorts occurred simultaneously and was only separated in analysis. Logistic regression was carried out to predict BRCA versus negative in a model with TC scores, as well as non‐BRCA versus negative. Area under the receiver operating characteristic (ROC) curve (AUC) was calculated to assess model fit.

**Results:**

This study found an average TC of 7.71%. A family history of cancer was noted in 78.30% of patients, and a personal history of cancer other than breast occurred in 20.74% of patients. The presence of PVs and VUS was evaluated, and 12.03% of patients were found to have a PV, with an average TC of 8.98%. The most common PVs were CHEK2, BRCA2, BRCA1, and BARD1. Out of those with PVs, 52% had non‐BRCA1/2 PVs with an average TC of 5.47%. A total of 102 patients (10.58%) had a VUS, with an average TC of 8.29%. In further statistical analysis, TC were distributed significantly differently among the three groups, with differences observed between the BRCA group and negative group, as well as between BRCA and non‐BRCA1/2 PVs group. A higher TC was also associated with BRCA1/BRCA2 variants compared to non‐BRCA1/2 PVs.

**Conclusion:**

TC scores provide valuable information regarding the lifetime risk of an individual of developing breast cancer. However, the study found they were not associated with prediction of non‐BRCA1/2 PVs. When choosing a genetic testing panel for breast cancer genes, TC is not as a reliable predictor on individual patient’s family history, NCCN guidelines, or ASBrS guidelines. Our study supports the need to develop a genetic risk calculator that incorporates the predictive value for these non‐BRCA1/2 PVs in otherwise low or average TC women.

## 1. Introduction

Breast cancer is a significant public health concern, affecting millions of women worldwide. It is the most common cancer among women and the second leading cause of cancer‐related deaths in women globally [1]. Advances in screening and management have improved survival rates, but early detection remains the key to successful treatment. The identification of high‐risk women who may benefit from increased surveillance or prophylactic measures has become increasingly important. The Tyrer–Cuzick (TC) model is a risk prediction tool designed to estimate the likelihood of developing breast cancer in unaffected individuals, integrating both family history and hormonal risk factors.

The TC Lifetime Risk Score is a widely used tool for predicting the likelihood of developing breast cancer in women. It is based on a range of factors, including age, family history, and reproductive history, and has been shown to be highly accurate in predicting the risk of breast cancer in both the general population and in women with a family history of the disease [2]. The TC model incorporates more than 20 factors, including age at menarche, age at first live birth, number of first‐degree relatives with breast cancer, and presence of atypical hyperplasia, among others [2]. Of note, the TC risk model is not suited for patients that have a personal history of breast cancer, as it only estimates the risk of developing primary breast cancer in unaffected individuals.

Recent studies have investigated the ability of the TC to predict the risk of developing breast cancer in women with pathogenic variants (PVs) in genes, such as BRCA1/2. Evans et al. report an AUC of 0.59 for BRCA1 and 0.61 for BRCA2 carriers using the TC model, suggesting poor discriminatory ability in high‐risk populations [3]. Kuchenbaecker et al. highlight limitations in TC model calibration when applied to mutation carriers, reinforcing its limited utility for this population [4]. These genes are well known to be associated with an increased risk of breast and ovarian cancer, and identifying carriers is crucial for implementing appropriate screening and prevention strategies. The accuracy of the TC Lifetime Risk Score in predicting breast cancer risk in carriers of PVs is of particular interest, as it may help to identify those who would benefit from enhanced surveillance or prophylactic measures. The objective of this study is to assess the strength of association of the TC model with BRCA1/2 and non‐BRCA PVs.

## 2. Materials and Methods

### 2.1. Data Collection

The Medneon iGAP Registry is a comprehensive database that contains clinically annotated data, specifically designed to capture the use of germline genetic, genomic, and other biomarker testing in routine clinical practice. The registry records a wide range of patient‐related data such as age, sex at birth, ethnicity/race, zip code, relevant disease diagnoses, and other clinical history. Additionally, it captures information on germline genetic, genomic, and other biomarker testing results, treatment plans, follow‐up plans, and short‐ and long‐term outcomes data. Enrollment in the registry was conducted via various breast surgeons, breast oncologists, and primary care physicians, including not only high‐risk patients but also those that fall into the population tested for breast cancer. Patients were excluded from the patient population based on lack of data for calculation of the TC score or refusal of enrollment in the study. The board of clinical advisors, comprising physicians, nurses, and patient advocates, who are active users of germline genetic, genomic, and biomarker testing, designed the Registry’s data collection process. This study was approved by the Institutional Review Board of Northwell Health (21‐1136).

### 2.2. Data Extraction

The registry data extraction was completed in October 2022 from 21 active research sites, at which point there were a total of 3769 subjects enrolled in the registry. These subjects were analyzed for completed data entry and data required for this report, especially the TC. 964 subjects (26%) of enrolled subjects had a completed data record including the germline genetic test report, TC, and cancer diagnosis information. It was an actively enrolling study at the time of patient selection and data extraction; as such, only complete data was included for analysis. Patients were followed from time of enrollment until 2022. Among the 963 patients, 133 (13.8%) developed breast cancer, while others had non‐breast cancers including ovarian (*n* = 6), pancreatic (*n* = 2), and prostate (*n* = 1).

### 2.3. Statistical Analysis

TC scores were compared between the three patient cohorts that had BRCA gene mutations, non‐BRCA PVs, and negative for the aforementioned gene mutations, using the Kruskal–Wallis test followed by pairwise comparison using Dwass–Steel–Critchlow–Fligner (DSCF) adjustment for multiple comparisons. Logistic regression was carried out to predict BRCA versus negative in a model with TC, as well as non‐BRCA versus negative. The area under the receiver operating characteristic (ROC) curve (AUC) was calculated to assess model fit. AUC values were interpreted as follows: 0.5–0.6 = poor, 0.6–0.7 = fair, 0.7–0.8 = acceptable, per standard ROC interpretation criteria [5].

## 3. Results

**Figure 1:**
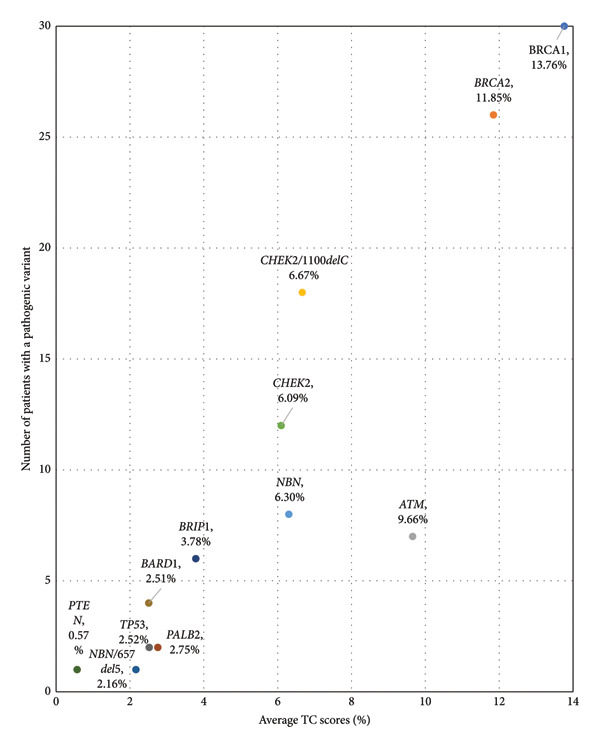
PVs are assessed based on their prevalence in our population, as well as the average TC score of the patients with this PV.

**Figure 2:**
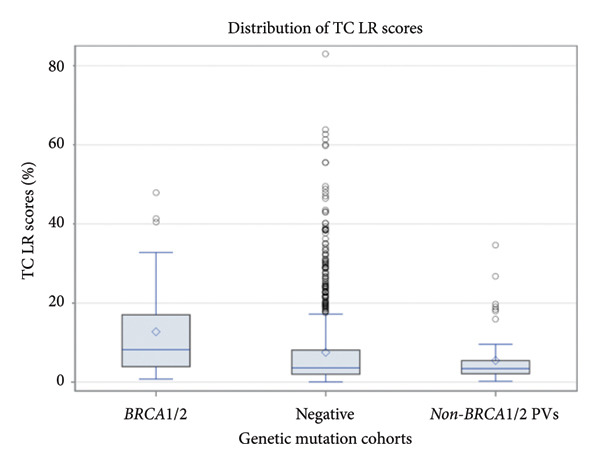
Distribution of TC within the among the three study cohorts including the BRCA1/2 group, the non‐BRCA1/2 PVs group, and the negative genetic mutation group.

A total of 964 patients with TC Version 8 scores were evaluated in this study. The average TC for the population was 7.71%. A family history of cancer was noted in 78.30% of patients, and a personal history of cancer other than breast occurred in 20.74% of patients. Historical TC were included for patients who eventually developed breast cancer in the three cohorts. Out of the total population, 133 patients (13.80% of the enrolled patient population) went on to develop primary breast cancer after their enrollment in the study. Figure 1 displays TC by gene for patients with PVs only, not including VUS. TC were distributed among the three study cohorts including the BRCA1/2 group, the non‐BRCA1/2 PVs group, and the negative genetic mutation group (Kruskal–Wallis *p* < 0.001) (Figure 2). The DSCF method for pairwise multiple comparison indicates that there were differences in the distribution of TC scores between the BRCA group and the negative group (*p* < 0.0001), as well as between BRCA and non‐BRCA1/2 PVs group (*p* < 0.0001). We fail to observe a significant difference in the distribution of TC between the non‐BRCA and negative groups (*p* = 0.572). When predating BRCA versus negative using TC alone, we observed AUC 0.691, demonstrating a significant value. When predicting the non‐BRCA1/2 PV group versus the negative group using TC alone, we observed an AUC of 0.539, demonstrating a failed value. AUC results have been considered fair for values between 0.7 and 0.8 and failed for values between 0.5 and 0.6. A higher TC was also associated with BRCA1/BRCA2 variants compared to non‐BRCA1/2 PVs (*p* = 0.038), using chi‐square analysis.

Demographic characteristics of the iGAP study cohort are summarized in Table 1. The majority of participants were geographically distributed across California (40.35%), Texas (23.03%), and Illinois (17.43%), reflecting the regions most represented in the registry. The racial composition was predominantly Caucasian (69.92%), followed by individuals identifying as Hispanic (5.60%), African/Black (3.63%), and Asian (2.49%), with 15.35% categorized as unknown. The age distribution demonstrated a balanced spread across decades, with the largest proportion of participants between 50 and 59 years (23.96%), followed by 40–49 years (22.93%) and 60–69 years (17.43%). When stratified by age, younger participants (< 55 years) demonstrated a markedly higher average TC (12.74%) compared to those aged ≥ 55 years (5.47%), aligning with the expected risk pattern in premenopausal populations (Table 2). These findings support the age sensitivity of the TC model, reflecting higher modeled risk estimates among younger women, who also represented the demographic most likely to carry high‐penetrance variants.

**Table 1:** Demographic information of iGAP study cohort, including geographical distribution, race distribution, and age distribution.

**Demographics of iGAP study cohort**		

	**Subjects (n)**	**Cohort (%)**

*Geographic distribution*		
California	389	40.35
Texas	222	23.03
Illinois	168	17.43
New Mexico	82	8.51
Michigan	56	5.81
Tennessee	15	1.56
Maryland	13	1.35
New York	11	1.14
Florida	8	0.83

*Race distribution*		
Caucasian	674	69.92
Unknown	148	15.35
Hispanic	54	5.60
African/Black	35	3.63
Asian	24	2.49
Ashkenazi	18	1.87
Other	10	1.04
Multiple	1	0.10

*Age distribution*		
< 30	53	5.50
30–39	173	17.95
40–49	221	22.93
50–59	231	23.96
60–69	168	17.43
> 70	118	12.24

**Table 2:** Age distribution information of iGAP study cohort.

Age	< 55 years old	≥ 55 years old
Number of subjects (*n*)	12.02%	8.57%
Average TC	12.84%	5.25%
Total average TC	**12.74%**	**5.47%**

*Note:* Specific age ranges allude to ages at testing roughly approximating menopause as ages less than 50 or ages greater than or equal to 50, due to the impact of this factor on genetic risk. The bold values are the total average.

Among the total cohort, 133 patients (13.8%) developed a primary breast cancer after enrollment, allowing for correlation between baseline TC estimates and subsequent disease onset. Across genetic subgroups, TC distributions were examined in three cohorts: BRCA1/2 PV carriers, non‐BRCA1/2 PV carriers, and genetically negative individuals. Figure 1 illustrates the prevalence of each PV and its corresponding mean TC, demonstrating that BRCA1 and BRCA2 mutations were the most prevalent and consistently associated with elevated TC scores relative to other genes. As shown in Figure 2, the distribution of TC scores differed significantly across cohorts (Kruskal–Wallis *p* < 0.001). Post hoc analysis using the DSCF method revealed significant pairwise differences between the BRCA1/2 group and the negative cohort (*p* < 0.0001) as well as between the BRCA1/2 and non‐BRCA1/2 PV groups (*p* < 0.0001), while no significant difference was observed between the non‐BRCA1/2 PV and negative cohorts (*p* = 0.572). ROC analysis further evaluated the discriminatory ability of TC scores for identifying germline mutation carriers. When differentiating BRCA1/2 carriers from genetically negative individuals, the TC model yielded an AUC of 0.691, indicating moderate predictive capacity. In contrast, prediction of non‐BRCA1/2 PV carriers versus negative individuals yielded an AUC of 0.539, denoting poor discrimination. According to conventional AUC interpretation, values between 0.7 and 0.8 are considered fair, whereas 0.5–0.6 reflect failed performance.

These results suggest that TC retains modest predictive ability for high‐penetrance BRCA1/2 mutations but performs suboptimally for lower‐penetrance variants. Finally, chi‐square analysis confirmed that higher TC scores were significantly associated with BRCA1/2 PVs compared to non‐BRCA1/2 PVs (*p* = 0.038). When stratified by breast cancer outcome, patients who developed breast cancer demonstrated elevated mean TC scores compared to those who remained unaffected, particularly within the BRCA1/2 subgroup (Table 3). Together, these findings indicate that the TC model maintains partial discriminatory power for BRCA‐related risk but is less effective in distinguishing non‐BRCA PVs, underscoring the need for model recalibration in genetically heterogeneous populations.

**Table 3:** Average TC of patients in the iGAP population, including those with BRCA, non‐BRCA PVs, and no PV.

**Average TC LR by germline result and breast cancer status**					
	**BRCA1/2 (%)**	**Non-BRCA1/2 PV (%)**	**Negative (%)**	**Total average TC (%)**	**Number of subjects**

Breast cancer	12.02	8.57	14.47	**14.16**	**134**
No breast cancer	12.84	5.25	6.35	**6.66**	**830**
Total average TC	**12.74**	**5.47**	**7.53**	**7.70**	**964**

*Note:* The bold values are the total values.

## 4. Discussion

The limitations of TC in predicting breast cancer risk in carriers of non‐BRCA1/2 PVs are significant, and this finding underscores the need for additional research into more accurate risk prediction models. Although the TC has been shown to be useful in identifying patients at higher risk of developing breast cancer, it may not be sensitive enough to identify those with a moderate or lower risk of the disease. This may result in unnecessary medical interventions for patients who may not need them or inadequate risk assessment for those who are at increased risk.

As our findings indicate that patients with non‐BRCA1/2 PVs had low TC scores, the score is not entirely comprehensive in its ability to be used as a sole designator for further genetic testing for breast cancer development. The inability for breast cancers of an unknown PV to yield notable TC scores clearly displays a limitation of this model in preventative or prediagnostic testing. As such, the decision for genetic testing should not be influenced solely by the TC score but should be based on individual patient’s family history, NCCN guidelines, or the TC score, as it provides a more wholistic perspective on individual patients. TC scores do show accuracy in assessing risk in patients with known PVs, such as BRCA1/2, and thus, still hold merit in their assessment in those patient cases. A comprehensive approach that includes both clinical and genetic risk factors may provide more accurate risk assessment for patients [6]. In addition, ongoing research into novel genetic markers associated with breast cancer risk may lead to the development of more accurate risk prediction models. Our study demonstrated that patients who were enrolled in the registry long enough to develop breast cancer demonstrated higher TC scores on average than those who were not diagnosed. This finding suggests that the TC score still retains some efficacy in identifying patients at higher risk of developing breast cancer [7].

Despite the promise of the TC risk model in predicting breast cancer risk, there are limitations to its accuracy. Future directions of this study could also look at the population diversity of the sample that was used to develop the TC risk score, as the TC may underpredict patients that are from non‐European descent. Alternate models for estimating risk prediction have also been developed, such as the Gail model. The Gail model had been developed using data from various ethnic and racial groups and was noted to perform well, but it was still noted to potentially show inaccurate risk results in specific non‐White populations, including Black/African American women with previous biopsies, Hispanic women born outside of the United States, and Asian women [8–11]. Ongoing research is being conducted to provide additional validation for certain subgroups and to improve the model [8]. Although the majority of the cohort analyzed in this study self‐identified as Caucasian, the available dataset did not contain sufficiently granular or complete ancestry‐specific information to support recalculation of TC performance within a European‐only subset. Nonetheless, the racial and ethnic composition of the registry highlights the need for future studies to evaluate the calibration of the TC model in diverse populations, as ancestry‐specific differences may meaningfully influence risk prediction accuracy.

Breast cancer remains a significant public health concern, and accurate risk assessment is essential for effective prevention and management of the disease. While the TC is a widely used tool for breast cancer risk prediction, its limitations in predicting breast cancer risk in carriers of non‐BRCA1/2 PVs highlight the need for continued research into more accurate risk prediction models. A comprehensive approach that includes both clinical and genetic risk factors may provide more accurate risk assessment for patients.

## 5. Conclusion

Our study investigated the performance of the TC risk model as a breast cancer risk predictor in patients with non‐BRCA1/2 PVs. The TC risk model remains a useful tool for predicting breast cancer risk in certain patient populations, especially those with BRCA1/2 mutations, but its limitations must be considered when making clinical decisions. Those that have other non‐BRCA1/2 PVs may need further genetic testing or risk models to assess their probability of developing breast cancer. Our findings suggest that the TC score may not accurately capture the risk in patients associated with variants; other factors such as individual patient’s family history, NCCN guidelines, or ASBrS guidelines, may be stronger predictors. Our study supports the need to develop a genetic risk calculator that incorporates the predictive value for these non‐BRCA1/2 PVs in otherwise low‐ or average‐TC women as well as further exploration for why the TC model does better to predict BRCA1/2 vs non‐BRCA1/2 PVs.

## Disclosure

We have previously submitted this article to Annals of Surgical Oncology and Breast Cancer Research and Treatment journals. The article was not accepted to either, however, a preprint was published to Research Square in the revision process.

## Conflicts of Interest

The authors declare no conflicts of interest.

## Funding

No funding was received for this manuscript.

## Data Availability

Research data are not shared.
